# International sport governing bodies as agents of diffusion—The case of World Athletics

**DOI:** 10.3389/fspor.2022.1025023

**Published:** 2022-11-11

**Authors:** Mara Konjer, Henk Erik Meier, Jörg Krieger

**Affiliations:** ^1^Institute for Sport and Exercise Sciences, University of Münster, Münster, Germany; ^2^Department of Public Health—Sport Science, Aarhus University, Aarhus, Denmark

**Keywords:** diffusion of sports, international sport governing bodies, World Athletics, diffusion strategies, athletics

## Abstract

The current paper conceptualizes international sport governing bodies (ISGBs) as “agents of diffusion,” whose key strategic interest is in the broadest participation in their sports. Our research examines the impact of a specific diffusion strategy, adopted by World Athletics in 2008, which was essentially the decentralization of decision-making power to license athletics events, and which aimed to increase the sports' visibility and accessibility, especially in previously underdeveloped markets like Africa. We evaluate these efforts' impact by analyzing data from the season's bests lists of World Athletics from 2001 to 2019. Therefore, we employ multilevel regressions. The results are complex but instructive. We find that the efforts were of limited success especially in target regions. Still, the strategy inspired more countries to invest in both hosting new events and sending athletes to new disciplines. However, our results cast some doubt about the sustainability of these efforts. The need for a better conceptualization of relevant domestic factors becomes evident. Furthermore, we find that diffusion strategies, which do not offer material incentives, are of limited efficacy.

## Introduction

Sport has been depicted as one of the most successful Western cultural exports. The concept of competitive, specialized sports originated in Britain and conquered the world *via* different mechanisms of diffusion. However, the diffusion of sport is not a unidirectional process of homogenization but also of heterogenization. Sport's global development has been created out of multiple, intersecting correspondences between histories, sites and social formations [(1, 2); see also (3)]. Moreover, increased competitive intensity and limited resource endowments seem to recently force countries to specialize in certain disciplines (4, 5). Accordingly, fierce global competition seems to catalyze processes of heterogenization of national elite sports and might pose limits to the diffusion of Western sports.

The current paper builds on these debates but also draws attention to the fact that the international sport governing bodies (ISGBs), such as the International Olympic Committee (IOC) or the Fédération Internationale de Football Association (FIFA), represent agents of diffusion, whose key strategic interest is in the broadest participation of countries in their sports or disciplines. Since the preconditions for diffusion vary widely and the ISGBs possess different organizational capacities, they have employed diverse diffusion strategies. Here, we examine the specific decentralization strategy of World Athletics (WA) (known until 2019 as the International Association of Athletics Federations—IAAF), which aimed to contribute to the diffusion of athletics. It is important to emphasize that we are not the first to examine the WA's diffusion efforts. Connor and McEwan (6) have presented a highly critical account of previous WA's programs. They not only doubted the efficacy of these efforts but criticized them as a neo-colonial project imposing a “white man's burden” on developing countries.

We proceed as follows: We first present the concept of diffusion and summarize key findings of previous research. Then, we explore the role of the ISGBs as “agents of diffusion” and discuss the specific strategy of World Athletics. After we depict our methodological approach, we present a quantitative case study on the World Athletics' diffusion strategy. Finally, we discuss theoretical and practical implications.

## Theoretical background

### Diffusion theory and mechanisms

Diffusion is a major topic in several sub-disciplines of the social sciences (7). To avoid conflation with neighboring concepts, we start from a broad definition:

Diffusion reflects the spread of a practice or organizational structure within a social system and can be understood as both process and outcome. As a process, diffusion is important because it captures causal associations among external and internal determinants in a system, or in concrete terms, from a source to an adopter. As an outcome, diffusion is often considered less interesting, as the increased incidence of most things is arbitrary and does not reflect any form of contagion or communication [(8), p. 30].

Research on diffusion processes has been particularly interested in identifying the mechanisms of diffusion. In a key contribution, DiMaggio and Powell (9) distinguished between coercive pressures, which result from power relationships and resource dependencies, mimetic pressures, which refer to the adoption of widespread practices by default to reduce search costs, and normative pressures, which are defined as need to comply with the norms collectively shared in the organizational field (10). Since these rather social constructivist concepts are not related to the objective consequences of a practice (7), scholars of policy diffusion emphasize the relevance of learning and competition as mechanisms. While learning is motivated by the consequences of similar policies in other units, competition occurs when units react to one another to attract or retain resources (11).

### Mechanisms behind sports' diffusion

According to Guttmann (12), modern sports, originated in Britain, differed from other forms of physical culture by their secular, specialized, competitive, and record-seeking character. Although research on the diffusion of sports has not always clearly distinguished between the diffusion of sports as physical activity, as social structure, or as elite sports policies, it is uncontroversial that sports' early diffusion related to the economic and political dominance of the British empire (3, 13), which coincided with the first wave of globalization at the turn of the twentieth century (14).

We can categorize four underlying mechanisms and waves that drove the diffusion of sports:

(1) Coercive pressure during colonialization

The initial diffusion of sport relied substantially on coercion. British imperialists used sports as an instrument for transferring British moral values and for exerting cultural control (13, 15).

(2) Mimetic pressure by globalization

Sport diffused, however, also by mimetic processes. British diplomats, merchants, entrepreneurs, and sailors brought their pastimes to other European countries and international trading places and encouraged imitation (3, 16).

(3) Normative pressure by the British Empire

Moreover, the reputation of the Empire inspired other nations to emulate the British by adopting their pastimes and games (3, 16).

(4) Mimetic and normative pressures in the “world polity” after the Second World War

Similar imitative and normative pressures seem also to account for the spread of elite sports after the Second World War, which appears to have been an element of the broader process of the diffusion of concepts of Western statehood in a “world polity” (17). World polity theory claims that the Second World War inspired the emergence of a more global social order relying on cultural scripts:

As centralized nation-state solutions to social problems (including a world state) became less feasible, cultural emphases were reconstructed to ground a wide range of societal goals, such as protecting human rights or the natural world, in a way that would have seemed the duty of hierarchical empires or strong national governments in previous eras [(18), p. 368].

World polity theory represents a global-level application of sociological neo-institutionalism according to which organizations are shaped by external cultural expectations rather than by functional requirements (19). Hence, the world polity is supposed to rely on widely held cultural scripts about progress, justice, development, and human rights, as well as the structure and activities of nation-states:

Many features of the contemporary nation-state derive from worldwide models constructed and propagated through global cultural and associational processes [(17), p. 144–145].

Elite sport policies appear to be one element of the propagated Western nation-state model in particular because many international sport competitions are based on the principle of national representation that reinforces a strong association between modern statehood and international elite sports (20). Therefore, “international sporting competitions function so effortlessly as metaphor for the state of the nation at the popular political level” [(21), p. 285]. New nation states have quickly applied for recognition by the International Olympic Committee (IOC) or the Fédération Internationale de Football Association (FIFA). Thus, the de-colonialization after the Second World War resulted in a rapid growth of membership in these ISGBs (22).

### Barriers to the future diffusion of elite sports policies

Sport's global development has, however, not been subject to a simple logic of adoption and imitation (3). The history of modern sports provides examples of failed (16, 23) or reversed diffusion (3). Hence, the diffusion of sports is shaped by a continuous interplay of the global and the local (2, 24). More recently, scholars have emphasized that smaller countries have started to specialize in certain sports or disciplines, which comes with implications for sports' diffusion. The trend toward specialization seems to reflect strategic efforts of countries to better target scarce resources for elite sport development (25, 26). For example, smaller and poorer countries face pressure to focus on sports and disciplines with lower technical and infrastructure requirements (27–29). The impact of such specialization efforts is indicated by macro-social research on determinants of athletic success. Scholars have assumed that a country's population size defines its talent pool, while the state of economic development provides the opportunities to develop these talents (30). Such simple macro-social approaches have performed quite well in explaining success at the Olympics or in international soccer [e.g., (31)]. Recently, the predictive power of these models has declined (32). Moreover, Olympic medal winners have become more diverse and less predictable (29). In some disciplines, dominant nations have changed (5, 33). Here is relevant that such specialization strategies imply inevitably that some countries will choose not to or cannot adopt certain sports. Hence, the heterogenization of national elite sport systems might impede the diffusion of (some) sports.

### International sport governing bodies as “agents of diffusion”

The diffusion of Western models relies, however, not exclusively on cultural scripts but has also been supported by an “organizational apparatus” (17), which includes international and non-governmental organizations (18). In international elite sports, the ISGBs represent such an organizational apparatus. The diffusion of their sports has been one of the ISGBs' traditional key missions. Moreover, during the second half of the twentieth century, many ISGBs turned into revenue maximizing event organizers and became heavily dependent on business models, which ultimately rely on the broad diffusion of the sports they represent [e.g., Eisenberg, (34)]. However, the successful commercialization of international elite sports allowed many ISGBs also to fund diffusion efforts, that is, to provide material incentives for joining the ISGBs and for developing national elite sport systems. Hence, the diffusion of sports relies not only on cultural scripts but also on organizations. However, the ISGB's capacities to provide material incentives differ considerably.

For a long time, therefore, the usual approach of ISGBs was to provide non-material rather than financial support, e.g., in the form of education programs for coaches. These measures have repeatedly been subject to strong criticism, especially from the developing countries themselves, which rightly questioned their efficiency and repeatedly argued for material and administrative support (35). In a more fundamental way, scholars have criticized that such diffusion efforts to “may serve to reproduce the corrosive, post-colonial donor-recipient divisions” (36). In particular Connor and McEwan (6) criticized that the diffusion strategies of the ISGBs impose a “white man's burden” on developing countries as they motivate them to invest in expensive elite sports systems rather than in policies targeting the general population. At the same time, Connor and McEwan (6) doubt the efficacy of these diffusion efforts. In the next section, we will present World Athletics' (WA) diffusion strategies, focusing in particular on a very specific measure of the early 2000s.

### World Athletics' specific diffusion strategy

As Krieger (35) depicts, as early as the 1970s, WA sought to support new member federations, particularly from Africa and Oceania, through outreach programs to increase participation in their sport. Due to limited financial resources, these programs initially consisted exclusively of non-material assistance in the form of coach education courses. Despite the beginning commercialization of the sport in the 1980s and the resulting greater financial opportunities, the support of member federations from developing countries by WA was limited almost exclusively to non-material support until the 2000s. The success of these efforts was repeatedly questioned, especially by African member federations, where the development of the sport also failed due to a lack of infrastructure and administration.

In the 1990s, athletics experienced its commercial and popular peak worldwide, which might account for the fact that the development of athletics in developing countries was not an organizational priority for the WA. This changed, however, when WA became increasingly concerned about the commercial future of athletics from the early 2000s on (37). Athletics faced growing competition in particular from football for media coverage and sponsorship. Moreover, the development of athletics in the distinct regions followed divergent paths. While athletics experienced a boom in Asia, European markets seemed to decline, and athletics still faced problems to root in Africa. In the 2010s, the IOC substantially increased subsidies from the Olympic Solidarity Program for National Olympic Committees (NOCs) for developing countries (38). The financial means from the Olympic Solidarity program are intended for specific purposes, e.g. to support the professionalization of the NOCs' administration, educational programs for coaches etc. Sports federations are forced to adhere to these specific requirements and therefore cannot spend the financial resources for other purposes (39). Hence, even though WA also experienced also an increase in revenues, the organization remained unable to meet member states' demand for direct support, e.g., in the form of facility construction (40). Given these limited resources, WA identified the decentralization of organizational governance as a solution to these challenges and thus decisively changed its diffusion strategy.

WA's World Plan for Athletics of 2003 took first steps to “mobilize regions and member organizations” for achieving the strategic aim “to remain number one sport for individuals in a changing world” [(40), p. 12–13]. The mobilization should serve the central rationale to promote the further diffusion of athletics by increasing the sports' visibility. Hence, key markets like Africa were to be defined “in which the sport needs to maintain and also increase recognition” and in which it should be ensured that elite events were conducted. Furthermore, the World Plan of 2003 suggested to develop competition formats, which promoted an emotional connection between the public and the sport (40). After the 2003 World Plan envisioned to empower Area Associations to develop tailor-made programs and events and to improve access to local sponsorship and public subsidies, WA started decentralizing powers. The 2009 World Plan was fully committed to the rationale that the Area Associations were better prepared to address local needs and included the empowerment of the Area Associations as new focus area (41). Still, the aim was to implement “an international competition program, which will give more visibility and power to our continental associations” [(42), p. 4]. Hence, the World Plan suggested that the Area Championships “are given enough importance and visibility so that they can grow,” that “in each Area a consistent competition system is developed and implemented,” and that “the recognition of Athletics in each Area can grow and that commercial rights are marketed successfully” (7). Therefore, in 2008 WA changed its rules for sanctioning competitions (43). Previously, the WA council had the exclusive right to determine whether member federations could stage WA events (37). It is important to know that only performances achieved at licensed WA events entitle athletes to qualify for international championships and to enter the official best lists. For sponsors and other partners, as well as for promising athletes, official WA events are therefore much more attractive. From 2009 on, the authority to license events was handed over to the six Area Associations, which was seen as a fra-reaching change in WA's power structure and governance:

President Diack has just taken a dramatic step into the future. […] The President's plan is that the 5 Area Associations are now to be liberated and encouraged to manage their own affairs around the globe (41).

However, this decentralization was not accompanied by increased financial support from WA. Nevertheless, this strategy had the consequence that all six Area Associations held events in the second highest competition category, that is, World Challenge, in 2010 for the first time in WA history (44). Again, the devolution of authority was intended to serve to raise additional resources for regional competitions in order to “universalize and decentralize major athletic events” (45).

In the light of the previous theoretical considerations, WA can certainly be characterized as “agent of diffusion,” which faced limited organizational capacities and highly divergent local prerequisites. While WA identified decentralization as a means to raise additional resources for athletics, it continued to follow a very elementary diffusion strategy according to which increased visibility of and accessibility to athletics would catalyze its further diffusion. Visibility and accessibility should first of all be raised by increasing the number of events in all Area Associations. The link between international sports, modern statehood and national identification was supposed to create either mimetic or normative pressures on public authorities to invest in the further development of athletics.

To evaluate whether the decentralization strategy of WA catalyzed a broader diffusion of athletics, we conduct a quantitative case study on this particular diffusion strategy. In accordance with our theoretical reasoning, we address the following key question:

RQ1: To what extent has the decentralization strategy of WA catalyzed a broader diffusion, visibility and accessibility of athletics in terms of the number of athletics events held in all Area Associations?

In addition, we are interested in whether the decentralization strategy has inspired countries to organize events in athletics disciplines that are new to them. The assumption behind this is that it might be more difficult for some countries in the Global South to offer events, which require a specific and expensive infrastructure, such as pole vaulting. Therefore, it seems reasonable that the decentralization strategy therefore has only a limited impact on countries from the Global South. Accordingly, our second research question is:

RQ2: To what extend did WA's diffusion strategy inspire countries to start hosting events in new disciplines?

While increasing the visibility and accessibility of athletics through more events, especially in still underdeveloped countries of the Global South, was one of the primary goals of the strategy, WA was of course also keen to increase active participation in these events, and thus in global athletics as a whole. Thus, our third research question is:

RQ3: To what extend did WA's decentralization strategy inspire countries to start bringing athletes into disciplines of these events?

Besides that, the organization's diffusion strategy would only be considered successful if it had sustainable effects. Commenting on earlier diffusion efforts by the WA, Connor and McEwen (6) doubted such effects due to the lack of grassroots participation and infrastructure in the Global South. Thus, our fourth research question is:

RQ4: To what extend did WA's decentralization strategy inspire a persistent expansion of national elite sport systems?

## Research design and methods

Our research represents a quantitative case study since we examine “a contemporary phenomenon within its real-life context [where] the boundaries between a phenomenon and context are not clear and the researcher has little control over phenomenon and context” [(46), p. 14]. The case selection is “phenomenon-driven” (47) as it is motivated by WA's adoption of a new diffusion strategy in 2008. The strategy change allows applying what Yin (46) has called a “pattern matching logic,” that is, a comparison of the diffusion of athletics before and after the strategy change.

Our case study is not representative for all diffusion efforts by ISGBs. On the one hand, the preconditions for successful diffusion might be much more favorable for other sports. On the other hand, World Athletics implemented a very specific diffusion strategy. Nevertheless, the case study is theoretically significant because athletics is clearly a cultural export of the British and its diffusion has faced in particular problems in Africa (48). Hence, our case study can contribute to the analytical generalization of a theory on sports' diffusion.

### Data sources

To address our research questions, we analyze data on athletic events deriving from the season's bests lists in international athletics in the period from 2001 to 2019, which have been retrieved from the website of WA (44). This data source was chosen because of two clear advantages: the season's bests lists include, first, performances that reach a certain minimum standard and that, second, were achieved at events certified by World Athletics. Since WA's strategy is also to certify more events so that more nations have a chance to appear in the bests lists, these lists provide a very representative and reliable source for this study. One disadvantage, however, is that best lists only include the best performances of a certain athlete in a specific year. Therefore, we cannot analyze additional participation in other events. An alternative data source would have been to look at the result lists of all licensed events. However, these lists are not available for the period under investigation. Another one is that not all athletes that participated in a certain event necessarily appear in the best lists because of the defined minimum standard. However, these minimum standards are so low that it can be assumed that a critical amount of athletes, even from developing countries, should easily reach these standards, especially because they have to reach them in officially licensed events. For example, the women's 100 m best list for 2022 contains 3,942 entries up to a minimum performance of 12.30 s, which is almost 2 s slower than the fastest woman that year.

### Dependent variables

For our quantitative analyses, we rely on four dependent variables. The first one is EVENTS_c, j, t_, which consists of the number of events a country (c) is hosting in a certain discipline (j) in a certain year (t). We use this to evaluate the overall impact of the decentralization strategy on visibility and accessibility (RQ1), since having more events in all Area Associations and countries was the key goal of WA's strategy. If EVENTS is increasing after 2008, the year of the implementation of the new decentralization strategy, we consider this to be an indication of the success of the strategy.

To test whether WA's strategy inspired countries to start hosting events in new disciplines (RQ2), we coded a binary dependent variable called HOSTENTRY_c, j, t._. A country (c) is coded as entering a discipline (j) with an event in a certain year (t) if a country's event appears on the season's best list after no event had appeared on the season's best the year before.

Our third dependent variable refers to the athletes participating in the events rather than the events themselves. Finally, a fundamental concern of all ISGBs is to increase participation in their sport. To evaluate whether the strategy inspired countries to start sending athletes to these events in new disciplines (RQ3), we coded another binary variable called ATHENTRY_c, i, t_. A country (c) is coded as entering a discipline (j) in a certain year (t) if a country's athlete appears on the season's best list after no athlete had appeared on the season's best the year before. It should be noted that not all athletes train in their countries of origin, which means that any developments in this area cannot be attributed solely to decisions made by one country.

The last dependent variable, which serves to answer RQ4, i.e., whether these efforts are persistent, is another binary variable (ATHEXIT_c, i, t_). A country's (c) exit of a discipline (j) in a certain year (t) is coded if no country's athlete appears on the season's best after at least one athlete had appeared on the season's bests list the year before.

Of course, as with ATHEXIT, we also wanted to test whether the strategy had a lasting effect on event alignment and we coded a dependent variable HOSTEXIT for this purpose. However, there was no case in which a country, after having started to host events in one discipline, gave up again. We can therefore anticipate: If WA manages to inspire countries to host events, they will stick with it.

Descriptive statistics of our dependent and independent variables are provided in Table 1.

**Table 1:** Dependent and independent variables.

**Name**	**Definition**	**Level**	**Type**	**Obs**	**Min**	**Max**	**Mean**	**SD**
**Dependent variables**								
EVENTS	Number of events that a country hosts in a discipline per year.	Discipline	Continuous	195,893	0	617	4.01	19.21
HOSTENTRY	1 = A country starts to host events in a certain discipline in that year, otherwise = 0	Discipline	Binary	195,893	0	1	0.459	0.498
ATHENTRY	1 = a country's athlete appears on the season's best list in a certain discipline after no athlete had appeared on the season's best the year before, otherwise = 0	Discipline	Binary	195,893	0	1	0.044	0.206
ATHEXIT	1 = no country's athlete appears on the season's best in a certain discipline after at least one athlete had appeared on the season's bests list the year before, otherwise = 0	Discipline	Binary	195,893	0	1	0.033	0.179
**Independent variables**								
**Level 1 predictors**								
STRATEGY CHANGE	1 = in years after 2008, otherwise = 0	Discipline	Binary	195,893	0	1	0.60	0.49
GENDER	A binary measure for men's and women's disciplines, 1 = men's discipline, 2 = women's discipline	Discipline	Binary	195,893	1	2	1.49	0.50
**Level 2 predictors**								
POPULATION		Country	Categorical					
Very small population	< 1 m inhabitants			195,893	0	1	0.18	0.39
Small population	1–5 m inhabitants			195,893	0	1	0.18	0.39
Low middle population	5–50 m inhabitants			195,893	0	1	0.48	0.50
Middle population	50–100 m inhabitants			195,893	0	1	0.08	0.28
Large population	>100 m inhabitants			195,893	0	1	0.07	0.26
GDP PER CAPITA		Country	Categorical					
Low income	< 995 USD			195,893	0	1	0.18	0.39
Middle income	995–3,895 USD			195,893	0	1	0.23	0.42
Upper middle income	3,895–12,055 USD			195,893	0	1	0.24	0.43
High income	>12,055 USD			195,893	0	1	0.34	0.48
FREEDOM	1 = Low degree of freedom; 7 = High degree of freedom	Country	Continuous	195,893	1	7	3.21	1.89
NOCAGE	Number of years since a country's National Olympic Committee has been officially recognized by the IOC	Country	Continuous	195,893	0	125	56.58	31.48
ASSOCIATION		Country	Categorical					
Africa	1 = Country belongs to Area Association of Africa, otherwise = 0			195,893	0	1	0.23	0.42
Asia	1 = Country belongs to Area Association of Asia, otherwise = 0			195,893	0	1	0.22	0.42
ConSudAtle	1 = Country belongs to Area Association of South America, otherwise = 0			195,893	0	1	0.06	0.25
Europe	1 = Country belongs to Area Association of Europe, otherwise = 0			195,893	0	1	0.29	0.45
NACAC	1 = Country belongs to Area Association of North America, otherwise = 0			195,893	0	1	0.13	0.34
Oceania	1 = Country belongs to Area Association of Oceania, otherwise = 0			195,893	0	1	0.06	0.24
**Control variables**								
TREND	Trend variable ranging from 1 to 19 for years 2001 to 2019	Discipline	Continuous	195,893	1	19	10.24	5.46
DISCIPLINE GROUP		Discipline	Categorical					
Sprint	1 = Discipline is 100, 200, or 400 m, otherwise = 0			195,893	0	1	0.16	0.36
Middle distance running	1 = Discipline is 800 or 1,500 m, otherwise = 0			195,893	0	1	0.10	0.30
Long distance running	1 = Discipline is from 3,000 m up to Marathon, otherwise = 0			195,893	0	1	0.14	0.35
Hurdles & Steeple chase	1 = Discipline is 100 or 110 m Hurdles, 400 m Hurdles or 3,000 m Steeplechase, otherwise = 0			195,893	0	1	0.15	0.35
Jumping	1 = Discipline is long jump, high jump, triple jump, or pole vault, otherwise = 0			195,893	0	1	0.20	0.40
Throwing	1 = Discipline is javelin throw, discus throw, hammer throw or shot put, otherwise = 0			195,893	0	1	0.20	0.40
Walk	1 = Discipline is 20 or 50 km walk, otherwise = 0			195,893	0	1	0.07	0.25

### Independent variables

#### Level 1 predictors—discipline level

The key independent variable on level 1 is a dummy, which assumes “0” for the period before 2008 and “1” for the period from 2009 to 2019 (STRATEGY CHANGE). Thus, the coefficients for this variable represent the impact on the dependent variables after the strategy change. Additionally, we coded another variable on discipline level to control for gender differences (GENDER). The displayed coefficient shows the impact of female gender on the dependent variables.

#### Level 2 predictors—country level

Since WA aimed in particular to diffuse athletics in Africa, we categorize the distinct Area Associations (ASSOCIATION) and include them in the model. Europe serves as basic category since athletics has its roots here. Additionally, we consider the existence of a national elite sport tradition as measured by the age of the first acknowledged National Olympic Committee (NOC) (NOCAGE). Following macro-social approaches we control for the strength of the national economy (GDP PER CAP) and its size in terms of population (POPULATION). As a proxy for basic features of the political system, we rely on the Freedom in the World Index provided by Freedom House (49) (FREEDOM).

#### Control variables

We calculated a trend variable (TREND) ranging from 1 to 19 for the years 2001 to 2019 to account for the longitudinal character of the data (50). To account for differences among athletic disciplines, they were combined into groups (DISCIPLINE GROUP).

### Analytic strategy

Our research design follows conventions of other macro-sociological approaches to elite sport development [e.g., (51)]. For conducting multivariate analyses, a data set with panel structure was created: it contains 195,893 observations for all 41 disciplines for the 217 countries in a certain year. The dataset has a two level-structure with athletic disciplines as level 1 units and countries as level 2 units. We report the coefficients for the level 1 and level 2 independent variables (see Table 1) and provide information on level variances and model fits. We tested whether the level 1 predictor effects (GENDER and STRATEGY CHANGE) varied across countries. If this was the case, random slopes were integrated (52).

We employed eight models to answer our research questions. In the first two models (1a and b) we analyze whether the number of hosted events (EVENTS_c, j, t_) was affected by the strategy change. Since EVENTS is a continuous variable, we could employ multilevel linear regressions. The first model only reports general results, while in the second model, we included interactions between STRATEGY CHANGE and our control variables to model how exactly the strategy change affected each member association, each with different conditions. For the other three dependent variables, we proceeded in the same way. However, because binary dependent variables were present in models 2 to 4, we applied multilevel logistic regression models here and report odds ratios instead of coefficients.

## Findings

We present our findings following our research questions.

### RQ1: Impact in terms of hosted events in the area associations

**Figure 1:**
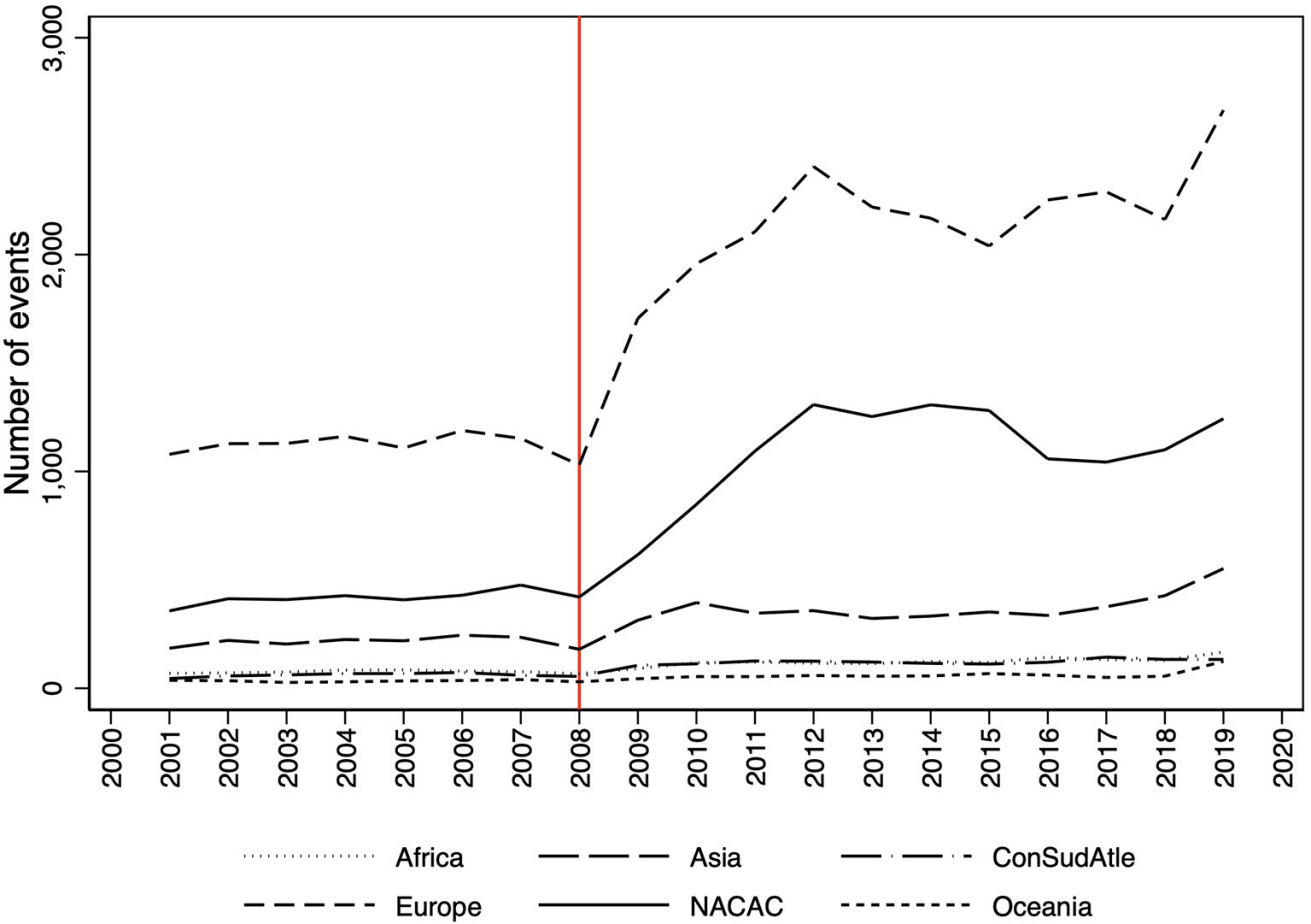
Development of the number of athletics events in the area associations.

The visual presentation suggests that WA's decentralization strategy had a strong positive impact (Figure 1). At least there is an increase of events for some of the Area Associations after 2008, mainly Europe and North America.

Before we now discuss the multivariate results (Table 2), it should be noted that the four interaction models (1b, 2b, 3b, and 4b) are particularly interesting for answering our research questions. Therefore, we will discuss the results of these models in more detail. Nevertheless, we will also briefly report the “general models” because they provide insight into basic developmental mechanisms in global athletics.

**Table 2:** Multilevel regressions.

	**EVENTS**		**HOSTENTRY**		**ATHENTRY**		**ATHEXIT**	
	**Model 1a**	**Model 1b**	**Model 2a**	**Model 2b**	**Model 3a**	**Model 3b**	**Model 4a**	**Model 4b**
**Independent variables**	**Coefficient (SE)**	**Coefficient (SE)**	**Odds ratios (SE)**	**Odds ratios (SE)**	**Odds ratios (SE)**	**Odds ratios (SE)**	**Odds ratios (SE)**	**Odds ratios (SE)**
**Level 1 predictors**								
STRATEGY CHANGE	1.237 (0.933)	−8.303** (2.814)	3.781*** (0.125)	0.594*** (0.085)	4.093*** (0.382)	14.688*** (4.739)	1.476** (0.179)	2.482^†^ (1.173)
**GENDER** ^**a**^								
Female	−0.006 (0.045)	0.007 (0.071)	1.052** (0.017)	1.048^†^ (0.028)	0.767*** (0.035)	0.710*** (0.043)	0.755*** (0.037)	0.870 (0.080)
**Level 2 predictors**								
**ASSOCIATION** ^**b**^								
Africa	−0.909 (0.790)	−0.610 (0.764)	0.052*** (0.035)	0.068*** (0.048)	1.133 (0.308)	0.952 (0.222)	0.994 (0.240)	1.351 (0.264)
Asia	−0.713 (0.794)	−0.942 (0.772)	0.395 (0.270)	0.335 (0.237)	1.819** (0.419)	1.268 (0.294)	1.230 (0.297)	1.725** (0.329)
ConSudAtle	−1.863 (1.138)	−1.775 (1.078)	0.256 (0.257)	0.110* (0.114)	1.192 (0.374)	1.059 (0.329)	1.106 (0.364)	1.459^†^ (0.329)
NACAC	0.489 (0.884)	0.390 (0.836)	0.100** (0.078)	0.123* (0.100)	1.357 (0.336)	1.183 (0.290)	1.060 (0.276)	0.778 (0.157)
Oceania	−0.285 (1.181)	−0.242 (1.113)	0.020*** (0.022)	0.011*** (0.012)	0.341** (0.128)	0.233 (0.091)	0.247*** (0.099)	0.639 (0.292)
**POPULATION** ^**c**^								
Small population	−0.068 (0.409)	0.272 (0.441)	1.324* (0.185)	1.004 (0.149)	1.741** (0.301)	1.315 (0.236)	1.336 (0.246)	0.859 (0.172)
Low middle population	−0.027 (0.493)	0.596 (0.533)	0.653* (0.115)	0.533** (0.101)	1.509* (0.282)	1.260 (0.237)	1.411^†^ (0.278)	0.598** (0.119)
Middle population	−0.354 (0.632)	0.981 (0.819)	0.592* (0.134)	0.498** (0.123)	1.130 (0.279)	0.962 (0.282)	0.994 (0.259)	0.525* (0.159)
Large population	0.465 (0.801)	4.780*** (1.168)	0.458** (0.119)	0.132*** (0.038)	1.219 (0.346)	1.394 (0.458)	1.097 (0.331)	0.574^†^ (0.180)
**GDP PER CAPITA** ^**d**^								
Middle income	−0.454** (0.164)	0.050 (0.210)	1.052 (0.052)	1.216** (0.073)	1.232** (0.096)	1.091 (0.115)	1.245** (0.105)	0.953 (0.145)
Upper middle income	−0.633** (0.216)	0.154 (0.281)	1.492*** (0.097)	1.200* (0.096)	1.496*** (0.148)	1.047 (0.139)	1.231^†^ (0.133)	0.688* (0.125)
High income	−0.388 (0.273)	0.605 (0.381)	2.127*** (0.193)	1.377** (0.147)	0.995 (0.127)	0.886 (0.148)	0.992 (0.141)	0.998 (0.264)
FREEDOM	−0.077 (0.066)	−0.153* (0.087)	0.885*** (0.018)	0.840*** (0.018)	0.887*** (0.025)	0.969 (0.032)	1.020 (0.031)	0.977 (0.548)
NOCAGE	0.061*** (0.010)	0.044*** (0.010)	1.082*** (0.009)	1.080*** (0.010)	1.003 (0.003)	1.006^†^ (0.003)	1.005 (0.003)	0.998 (0.471)
**Control variables**								
TREND	0.086*** (0.013)	−0.042* (0.019)	0.965*** (0.009)	0.945*** (0.010)	0.907*** (0.004)	1.035** (0.011)	0.967*** (0.005)	0.975 (0.022)
**DISCIPLINE GROUP** ^**e**^								
Sprint	5.286*** (0.105)	1.647*** (0.164)	52.568*** (2.434)	23.052*** (1.633)	2.096*** (0.126)	1.590*** (0.150)	2.091*** (0.147)	1.272 (0.287)
Middle distance running	3.698*** (0.113)	1.378*** (0.177)	22.200*** (1.068)	8.736*** (0.652)	1.995*** (0.127)	1.210^†^ (0.127)	2.276*** (0.167)	1.768* (0.427)
Long distance running	1.801*** (0.106)	0.699*** (0.165)	7.756*** (0.350)	2.999*** (0.212)	1.859*** (0.114)	1.110 (0.111)	2.086*** (0.148)	2.209** (0.512)
Hurdles & Steeple chase	2.136*** (0.106)	0.902*** (0.165)	17.593*** (0.802)	8.995*** (0.637)	1.548*** (0.096)	1.168 (0.115)	1.708*** (0.123)	1.268 (0.294)
Jumping	2.469*** (0.101)	0.845*** (0.158)	24.537*** (1.093)	14.347*** (0.986)	1.503*** (0.090)	1.099 (0.105)	1.580*** (0.111)	1.361 (0.306)
Throwing	2.026*** (0.102)	0.619*** (0.158)	21.052*** (0.936)	10.603*** (0.728)	1.247*** (0.076)	0.766** (0.073)	1.221** (0.088)	1.462 (0.341)
**Interactions**								
**STRATEGY CHANGE** **×GENDER**^**f**^								
Female		−0.020 (0.091)		1.006 (0.034)		1.095^†^ (0.058)		1.173 (0.131)
**STRATEGY CHANGE** **×ASSOCIATION**^**g**^								
Africa		0.256 (2.764)		0.586*** (0.048)		1.795* (0.431)		1.099 (0.274)
Asia		0.892 (2.778)		1.373*** (0.093)		1.964** (0.467)		0.754 (0.185)
ConSudAtle		−2.377 (4.071)		3.805*** (0.340)		1.345 (0.410)		0.492* (0.147)
NACAC		5.389^†^ (3.169)		0.713*** (0.045)		1.578^†^ (0.382)		1.719* (0.429)
Oceania		1.011 (4.225)		1.933*** (0.344)		1.729 (0.660)		1.721 (0.902)
**STRATEGY CHANGE** **×POPULATION**^**h**^								
Small population		−0.558 (1.041)		1.690*** (0.117)		0.778 (0.175)		0.661^†^ (0.161)
Low middle population		−1.232 (1.186)		1.269** (0.934)		0.501** (0.109)		0.780 (0.188)
Middle population		−3.031* (1.438)		1.406** (0.159)		0.488* (0.153)		0.893 (0.325)
Large population		−7.208*** (1.755)		4.152*** (0.543)		0.370** (0.129)		0.691 (0.349)
**STRATEGY CHANGE** **×GDP PER CAPITA**^**i**^								
Middle income		−0.612*** (0.340)		0.721*** (0.049)		0.938 (0.128)		0.946 (0.187)
Upper middle income		−0.689 (0.449)		1.275** (0.095)		1.060 (0.175)		0.948 (0.215)
High income		−0.707 (0.565)		1.081 (0.091)		0.559** (0.112)		0.998 (0.264)
STRATEGY CHANGE × FREEDOM		−0.114 (0.133)		0.975^†^ (0.015)		0.979 (0.041)		1.087^†^ (0.055)
STRATEGY CHANGE × NOCAGE		0.158*** (0.034)		1.002** (0.001)		0.985*** (0.003)		0.991** (0.003)
STRATEGY CHANGE × TREND		0.031 (0.039)		1.036*** (0.008)		0.865*** (0.010)		1.071** (0.027)
**STRATEGY CHANGE** **×DISCIPLINE GROUP**^**j**^								
Sprint		6.075*** (0.213)		4.426*** (0.404)		1.574*** (0.150)		0.158*** (0.046)
Middle distance running		3.930*** (0.230)		5.218*** (0.504)		2.165*** (0.287)		0.221*** (0.067)
Long distance running		1.907*** (0.215)		5.111*** (0.469)		2.207*** (0.280)		0.222*** (0.065)
Hurdles & steeple chase		2.127*** (0.214)		3.383*** (0.309)		1.582*** (0.201)		0.295*** (0.087)
Jumping		2.778*** (0.206)		2.726*** (0.241)		1.656*** (0.204)		0.184*** (0.053)
Throwing		2.417*** (0.206)		3.481*** (0.309)		2.120*** (0.270)		0.186*** (0.055)
Constant	−3.405*** (0.833)	−1.362 (0.828)	0.001*** (0.001)	0.004*** (0.003)	0.015*** (0.004)	0.013*** (0.004)	0.008*** (0.002)	0.857 (0.310)
**Variance components**								
Within-country (L1) variance	94.747*** (0.308)	94.102*** (0.306)						
Intercept (L2) variance	12.798*** (17.414)	11.222*** (1.154)	3.189*** (0.188)	3.286*** (0.194)	0.846*** (0.069)	0.822*** (0.070)	0.868*** (0.072)	0.249*** (0.104)
Slope (L2) variance STRATEGY CHANGE	175.177*** (17.414)	168.356*** (17.020)	–		1.105*** (0.071)	0.839*** (0.057)	1.480*** (0.095)	0.435*** (0.060)
Slope (L2) variance GENDER	–	–	–		0.490*** (0.042)	0.510*** (0.044)	0.501*** (0.045)	0.174*** (0.060)
**Additional information**								
ICC for null-model	0.447	0.447	0.832	0.832	0.226	0.226	0.221	0.221
AIC	14,015.62	14,002.81	95,729.25	94,349.59	62,837.84	62,454.97	10,300.1	10,247.05
Number of observations	189,375	189,375	189,375	189,375				
Number of groups	203	203	203	203	203	203	203	203
Number of observations per group (mean)	932.9	932.9	932.9	932.9	932.9	932.9	932.9	932.9

^a^Reference category is “Male”.

^b^Reference category is “Europe”.

^c^Reference category is “Very small population”.

^d^Reference category is “Low income”.

^e^Reference category is “Walk”.

^f^Reference category is “STRATEGY CHANGE = 1 × Male”.

^g^Reference category is “STRATEGY CHANGE = 1 × Europe”.

^h^Reference category is “STRATEGY CHANGE = 1 × Very small population”.

^i^Reference category is “STRATEGY CHANGE = 1 × Low income”.

^j^Reference category is “STRATEGY CHANGE = 1 × Walk”.

^***^p < 0.001, ^**^p < 0.01, ^*^p < 0.05,^†^p < 0.1.

The analyses for EVENTS (model 1) show an intra class correlation (ICC) of 0.447. Hence, almost 45 percent of the variance in this model, i.e., how many events are hosted per year by a country, can be explained by differences between the countries. This implies that we should look more closely at systematic and structural country differences, as, for example, in relation to economic or political differences. In addition, the tests showed that also the effect of STRATEGY CHANGE varied significantly across countries. Therefore, we applied a Random Intercept Random Slope-Model (RIRSM).

According to the general Model 1a, the STRATEGY CHANGE seemingly did not affect the number of events hosted by country. However, it is important to note that we allowed the effect of STRATEGY CHANGE to vary across countries, which could reduce a general effect. In contrast, the highly significant and positive coefficient for TREND indicates that the number of athletic events hosted by countries has been generally increasing over the entire period under investigation. As the coefficients for GDP PER CAPITA suggest, middle and upper middle-income countries generally seem to host less events than low-income countries. In contrast, a longer national sport tradition (NOCAGE) makes countries more likely to host events. The latter result is supported by some descriptive findings: The nations, which host the most events per discipline are still the traditional track and field nations, that is, the U.S. (156 per year), France (30), Germany (26), and Italy (22). Even rising nations like China (10) and Japan (13) are still lagging behind.

Modeling interaction effects (Model 1b) allows tracing which country conditions moderated the impact of the decentralization strategy. First of all, it becomes evident that the impact of WA's strategy did not vary much across the Area Associations (STRATEGY CHANGE × AREA ASSOCIATION) and between women's and men's disciplines (STRATEGY CHANGE × GENDER) since none of the coefficients is significant.

The negative and significant coefficients for the interactions with middle and large population countries (STRATEGY CHANGE × POPULATION) and for middle income countries (STRATEGY CHANGE × GDP PER CAPITA) have to be interpreted taken into account that the base categories are “very small population” and “low income.” Accordingly, the coefficients indicate that small and low-income countries were more likely to host more events after the implementation of the decentralization strategy. Furthermore, the strategic change inspired countries with a longer sport tradition to host more events (STRATEGY CHANGE × NOCAGE) as indicated by the positive and highly significant coefficient here. Moreover, sprint disciplines benefited most from the strategy change (STRATEGY CHANGE × DISCIPLINE GROUP). Thus, sprint events experienced the largest growth in events after 2008. All in all, our macroeconomic models do not seem to be able to comprehensively grasp the effect of decentralization. Both intraclass correlation and slope variance strongly suggest that member countries have responded very differently to the strategy, at least when it comes to the bare number of athletic events.

### RQ2: Impact on hosting in new disciplines

The answer to our second research question is slightly clearer. We report odds ratios. Odds ratios > 1 indicate that a variable increases the likelihood of HOSTENTRY, a variable with odds ratios < 1 decreases the likelihood, respectively. It is important to take into account that HOSTENTRY is a binary variable measuring whether a country has hosted an event for a specific discipline for the very first time. The ICC of 0.832 indicates that nearly 84 percent of the variance in starting to host events is explained by country differences. As the effect of STRATEGY CHANGE and GENDER varied significantly across countries, we again applied a Random Intercept Random Slope-Model (RIRSM). The general, Model 2a shows positive and highly significant odds ratios for STRATEGY CHANGE, meaning that WA's strategic change impacted the likelihood of starting to host events significantly. Moreover, women's events were slightly more likely to be hosted (GENDER) than men's events. Compared to the reference category Europe, countries of all other associations were less likely to start hosting athletics events in new disciplines (ASSOCIATION). HOSTENTRY was more likely to happen in small countries than in very small or bigger countries (POPULATION) and in richer countries than in low-income countries (GDP PER CAPITA), which would confirm our initial assumption that it is easier for countries with a good infrastructure to engage in new disciplines. The odds ratios smaller than 1 for FREEDOM suggest that authoritarian states were more likely to start hosting events. A look at the interactions (Model 2b) again allows us to analyze which country conditions moderated the impact of the strategy change. Thus, we see that after 2008 Asia, Oceania and especially South America seemed to be more inspired by the strategy than Europe, which serves as base category (STRATEGY CHANGE × ASSOCIATIONS), and more likely to start hosting events, but not Africa. Bigger and richer countries (STRATEGY CHANGE × POPULATION & STRATEGY CHANGE × GDP PER CAPITA) were more likely to start to host events than smaller and poorer countries after 2008. Moreover, countries with longer sport traditions were more affected by the decentralization strategy (STRATEGY CHANGE × NOCAGE) than countries with shorter sport tradition. The odds ratios for STRATEGY CHANGE × TREND indicate that the effect of the strategy is even increasing over time. Having a closer look into our data, however, we note that Nigeria is a notable exception in the African context: Despite being a low income-country, Nigeria has been involved in the hosting of events with new disciplines much more frequently than other countries since WA's strategy change. Since 2008, Nigeria has hosted 1,088 new events in new disciplines and is thus well ahead of the USA (877), France (875), and Germany (872). However, one country is not sufficient to influence general trends in the case of our macro sociological studies.

### RQ3: Impact on fielding athletes in new disciplines

To answer our third research question, we again employed multilevel logistic regressions with random intercepts and random slopes (RIRSM) for the dependent variable ATHENTRY, which measures whether a country is fielding an athlete in a new discipline for the first time in the period under investigation, allowing the effects of STRATEGY CHANGE and GENDER to randomly vary across countries. Nearly 23 percent of the variance in models 3a and 3b can be explained by country differences (ICC).

Model 3a suggests that the likelihood that countries send athletes to new disciplines increased significantly after STRATEGY CHANGE. There is, however, a higher likelihood for male athletes to enter new disciplines (GENDER) than for females. Furthermore, Asian countries seem to be much more and Oceanian countries much less likely than European countries to enter new disciplines (ASSOCIATION). Compared to athletes from countries with very small populations (POPULATION), athletes from rather middle-sized countries have a higher likelihood of entering, as well as athletes from countries with middle income in comparison to athletes from small income countries (GDP PER CAPITA). Again, less liberal countries are significantly more likely to send athletes into new disciplines (FREEDOM). In addition, there seems to be a general trend of decreasing likelihood that countries send athletes to new disciplines (TREND). Introducing interaction effects with STRATEGY CHANGE (model 3b) serves to qualify these findings. Compared to Europe, athletes from the member countries of all other Associations are more likely to enter new disciplines after 2008, which can certainly be explained by the fact that European countries have already been present in most of the disciplines before the WA changed its strategy. The significant odds ratios < 1 for the interactions of STRATEGY CHANGE with POPULATION, GDP PER CAPITA and NOCAGE suggest that World Athletics' decentralization strategy enabled smaller and poorer countries with shorter sport tradition to send athletes to new disciplines. Here, the countries with the highest number of new entrants are Tunisia, Singapore, Indonesia, Azerbaijan and Nigeria. The negative and significant coefficient for STRATEGY × TREND indicates, however, a declining impact of the strategy change over time.

### RQ4: Impact on persistent expansion of national elite sport systems

To answer our last research question, we analyzed whether athletes from a country exited from a discipline (ATHEXIT). Therefore, we again employed multilevel logistic regressions with random intercepts and random slopes (RIRSM), allowing the effects of STRATEGY CHANGE and GENDER to randomly vary across countries. Twenty-two percent of the variance is explained by country differences.

The basic model (4a) shows that an exit was more likely after STRATEGY CHANGE, which indicates an increased fluctuation. The risk to drop out of disciplines was smaller for female athletes than for male athletes (GENDER), which might indicate a more persistent impact of the strategy on women's sports than on men's sport. Interestingly, athletes from Oceanic countries were less likely to exit than athletes from European countries (ASSOCIATION). The odds ratios for GDP PER CAPITA indicate that athletes from countries with lower incomes are at greater risk to drop out than athletes from richer countries. The odds ratios for TREND demonstrate that the likelihood of dropping out is generally decreasing over time. Again, the inclusion of interactions effects serves to qualify these findings. The odds ratios for the interaction of STRATEGY CHANGE × TREND indicates that the risk of dropping out even increased after 2008. Besides that, most of the coefficients are no longer significant. However, we still see that being from a country with a longer sports tradition is related to a lower likelihood of dropout (NOCAGE). Furthermore, athletes from South American countries are less likely to disappear from the season's best, while athletes from North American countries are more likely (ASSOCIATION) than athletes from European countries. It should also be mentioned here that countries inspired by the change in strategy to field athletes in new disciplines for the first time (ATHENTRY) are now also among the countries most likely to drop out of disciplines again. These include Tunisia, Singapore, and Azerbaijan. Here, the strategy does not appear to have had a lasting impact. The situation is different here again for Nigeria, which records only a few withdrawals.

To summarize, the results of our analyses are complex. On the one hand, WA's strategy change did not significantly increase the visibility and accessibility of athletics in terms of the number of hosted events in the Area Associations of interest, especially in Africa. In addition, we found that while WA's decentralization strategy inspired Asian, South American, and Oceanian countries to start hosting events, this was less true for African countries with the particular exception of Nigeria. On the other hand, the strategy was more successful in terms of the actual goals, when it comes to sending athletes to new disciplines. Here, Africa and Asia in particular were more likely to field athletes in new disciplines than Europe or other associations. However, it must be noted here that this strategy can only be described as partially sustainable, as the risk of dropping out of disciplines again has increased overall after the change in strategy.

Besides these findings, it seems worthy to emphasize that female athletics experienced growth and diffusion in the period investigated. On the one hand, we have seen an overall increase in the number of events over the course of time, and on the other hand, the change in strategy has obviously had a lasting effect, in the sense that fewer women are dropping out of disciplines. However, as we found in another study, this effect is strongly dependent on the gender equality policies of the respective countries (53).

## Discussion

### Theoretical implications

The theoretical significance of our findings lies primarily in the limited success of World Athletics' decentralization strategy, which aimed to promote the diffusion of athletics. The strategy tried to rely on the traditional diffusion mechanisms of mimetic and normative pressure, which due to the association between modern statehood and participation in international elite sports account for the global spread of British sports. WA aimed at catalyzing diffusion by increasing visibility and accessibility of athletics by lowering requirements. The small but limited success of the top-down strategy in smaller and poorer countries and especially in Africa supports the claim that sport's future global development will be characterized by heterogenization, which will pose an enormous challenge for ISGBs. The strategy's limited sustainable effects can be interpreted as providing further evidence that countries more strategically target their investments in athletic performances. Small gains in international visibility and accessibility seem not to suffice to inspire more persistent efforts to invest in certain elite sports. Moreover, World Athletics' rationale that the decentralization of regulatory powers would mobilize additional local resources materialized primarily in already established or growing markets. Hence, a key implication of the case study is that increasing the visibility of a few elite athletes does not suffice to overcome the lack of grassroots in national physical culture and the challenge to meet the substantial infrastructure requirements. On a more fundamental level, the findings invite the speculation whether emulating Western elite sport systems has lost its appeal for countries in the Global South or whether the link between modern statehood and elite sport participation is weakening. In any case, developing countries seem less susceptive to further accept a “white man's burden” than critics assume (6).

Furthermore, the analyses are of theoretical relevance for a theory on sport's diffusion as they provide further evidence on facilitating factors. First, gender inequality affects diffusion. Countries are more likely to invest in male athletes. However, we could also see a trend of countries investing more in hosting female events. Here, however, it must be noted that this trend reflects the more general development of women's athletics: In the last 20 years, women's athletics has fundamentally developed, e.g., through new disciplines like hammer throw or 3,000 m hurdles. Accordingly, this trend might automatically increase the number of new events. Second, visibility and imitative behavior appear to be relevant. Sprint disciplines enjoy the broadest spread although they are overcrowded and success is rather unlikely. However, sprint disciplines are also characterized by the lowest infrastructure and equipment costs. Third, path dependencies and cultural resonance seem to matter. A longer tradition in Western sports serves to facilitate further adoption. Finally, domestic political regimes make a difference. Less liberal regimes might be more willing to invest in elite sports as a means to increase legitimacy and/or to be able to more easily mobilize the resources for elite sport development.

In sum, our theoretical contribution consists of providing further evidence for a heterogenization of sports' global development. In an increasingly diverse environment, resource limitations, cultural resonance, grassroots support and domestic political rationales will be more relevant for the diffusion of sports.

### Practical implications

Our findings come with practical implications for the agents of diffusion as well as for potential adopters. Concerning the ISGBs, the findings suggest that lowering infrastructure requirements can increase the visibility of sports as well as countries' willingness to host events. While the ISGBs can manipulate these target variables, such low-cost policies might not inspire a sustainable investment in national elite sport systems. Accordingly, in order to promote persistent diffusion, the ISGBs face the challenge to stronger subsidize elite sport development and to intensify efforts to inspire sustainable grassroots development.

Potential adopters should consider the fact that increased visibility of smaller countries does not necessarily affect the competitive hierarchy. Hence, poorer and smaller countries seem ill-advised to invest in elite sports policies in case there is no “trickle down” effect in terms of stronger grassroots participation. Otherwise, elite sport diffusion serves primarily the commercial aims of the ISGBs (6).

### Methodological limitations

It is important to note that we did not perform a strict evaluation of World Athletics' decentralization strategy as there is no control group. Some of the observed trends could also be due to other factors, such as the increase in scholarships awarded by U.S. universities to athletes from the Global South or the increased subsidies from the Olympic Solidarity Program to NOCs from the Global South. In particular, South American and African countries have benefitted from the increase in IOC subsidies since 2005 (38). However, even when such factors are taken into account, the development of athletics in particular in Africa appears to lag behind. Moreover, macrosocial approaches only scratch the surface of possible reasons for non-participation. Therefore, more “inside-out” research is needed to completely understand why strategies implemented by World Athletics have an impact on some countries and not have on others. Nigeria, for example, has stood out as the African country with the strongest development since the implementation of WA's decentralization strategy. Further qualitative research would be needed here to understand, which political decisions have led to this development and what has happened in this country since 2008 in order to draw further lessons for sports development. Another major limitation of the current study is that the strategies of the Area Associations have not been examined in detail.

## Data availability statement

Publicly available datasets were analyzed in this study. This data can be found at: https://worldathletics.org/.

## Author contributions

All authors listed have made a substantial, direct, and intellectual contribution to the work and approved it for publication.

## Conflict of interest

The authors declare that the research was conducted in the absence of any commercial or financial relationships that could be construed as a potential conflict of interest.

## Publisher's note

All claims expressed in this article are solely those of the authors and do not necessarily represent those of their affiliated organizations, or those of the publisher, the editors and the reviewers. Any product that may be evaluated in this article, or claim that may be made by its manufacturer, is not guaranteed or endorsed by the publisher.
